# Supplementary material for: Cryo-EM of prion strains from the same genotype of host identifies conformational determinants
Source: PLoS Pathog. 2022 Nov 7;18(11):e1010947. doi: 10.1371/journal.ppat.1010947 (PMC9671466; doi:10.1371/journal.ppat.1010947)
Supplement: S1 Fig — (A) Representative 2D class averages showing lateral views of a22L fibril segments. The enlargement of the class average boxed in green allows easier visualization of the fine horizontal bands running perpendicular to the fibril axis. (B) Fast Fourier transform of 2D class average boxed in green indicating signals at ~5.0 Å. (C) Fourier shell correlation plots of masked and unmasked models. (PDF) [file ppat.1010947.s001.pdf]

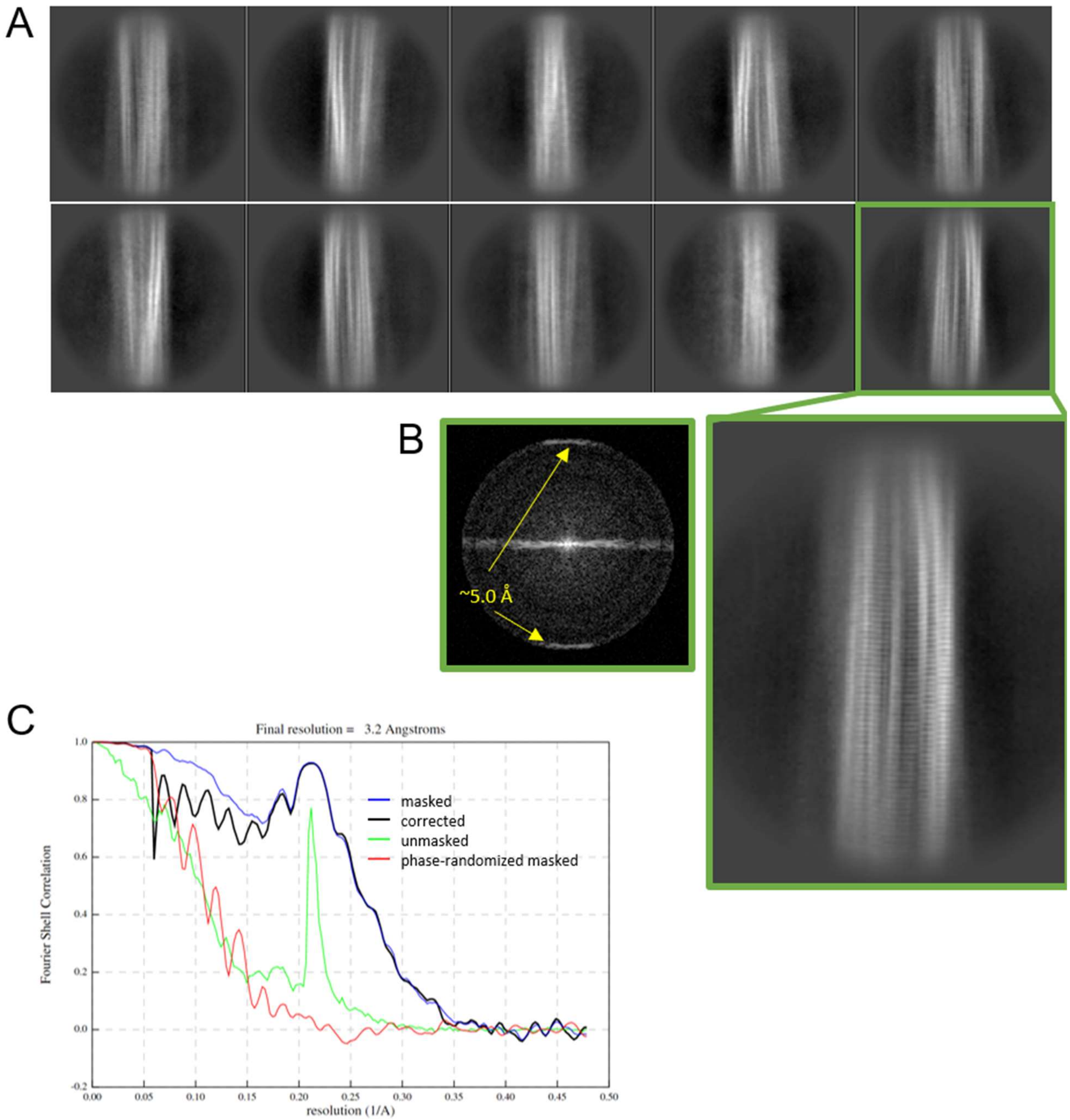

S1 Fig. (A) Representative 2D class averages showing lateral views of a22L fibril segments. The enlargement of the class average boxed in green allows easier visualization of the fine horizontal bands running perpendicular to the fibril axis. (B) Fast Fourier transform of 2D class average boxed in green indicating signals at ~5.0 Å. (C) Fourier shell correlation plots of masked and unmasked models.
