# Supplementary material for: Cryo-EM of prion strains from the same genotype of host identifies conformational determinants
Source: PLoS Pathog. 2022 Nov 7;18(11):e1010947. doi: 10.1371/journal.ppat.1010947 (PMC9671466; doi:10.1371/journal.ppat.1010947)
Supplement: S2 Fig — Compare to a22L in Fig 2D. (PDF) [file ppat.1010947.s002.pdf]

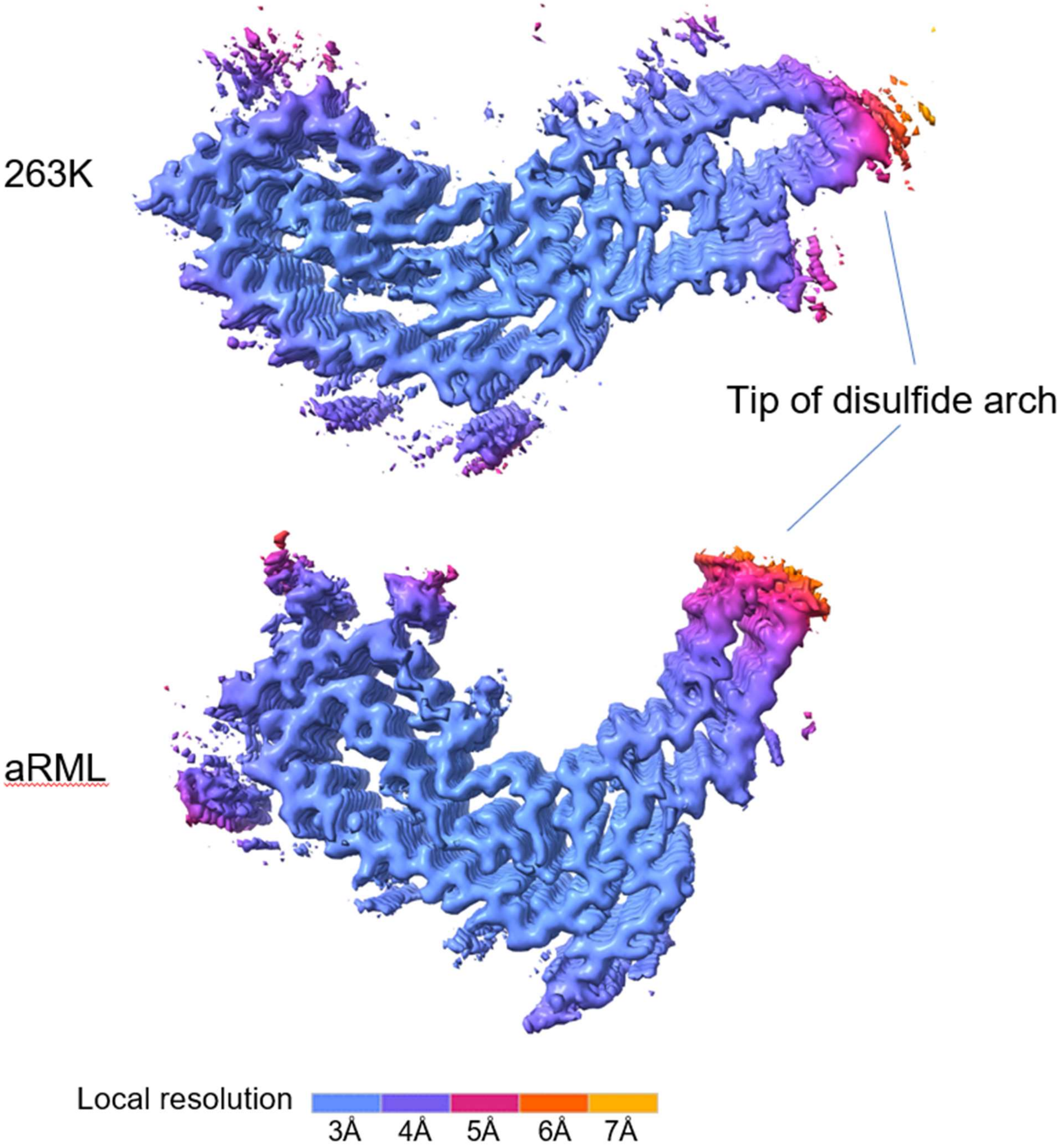


**S2 Fig.** Surface depictions of density map cross-sections of 263K (6) and aRML (5) prion fibrils with colors showing local resolutions according to the color bar. Compare to a22L in Fig. 2D.
