# Supplementary material for: Cryo-EM of prion strains from the same genotype of host identifies conformational determinants
Source: PLoS Pathog. 2022 Nov 7;18(11):e1010947. doi: 10.1371/journal.ppat.1010947 (PMC9671466; doi:10.1371/journal.ppat.1010947)
Supplement: Attachment — Submitted filename: Response to reviewers.docx [file ppat.1010947.s003.docx]

Dear Dr. Woerman,

Thank you and the reviewers for being positively disposed to our manuscript. We have revised the manuscript in response to the reviewers’ helpful comments as described point-by-point below. First, we would like also to point out a few other minor modifications that we have made:

1. We now define a22L as anchorless 22L in the Abstract.

2. We’ve come to realize that “β-arch” too specific a descriptor for a couple of the motifs that we had labeled as such in the a22L structure, so have backed off throughout the text and figures to the more general term “arch”. We also have added a note to this effect in the Introduction.

3. We have updated our ongoing bioassay results in the first paragraph of Results, which provide further confirmation of very high infectivity in our purified a22L prep.

4. We have reformatted the manuscript, figures, and supplement as stipulated for revisions in the instructions to authors.

Responses to reviewers (line numbers in our responses refer to those in the tracked revised manuscript):

Reviewer #2: *For Figure 2, the arrowheads have a fairly thick shape outline and the color differences weren't quickly obvious until I moved the image to a big screen. In addition, they may be difficult for someone who is RG colorblind.
line 324-328: it's hypothesized that the glycans can impose flexibility in the disulfide arch. it's also stated that the top third of the arch has locally low/moderate resolution of 5-7 Angstroms. This makes sense, but the previous paragraph (308-310) that the different disulfide arch conformations are the "most obvious differences between the ... strains." I don't doubt for a second that these are different strains, but it would be extremely helpful if the authors would help with the interpretation of the differences for those who are not structural biologists. if the conformational of the arches are flexible and not well resolved, how much confidence should the reader have that the three strains are dramatically different in this particular area? what is the most scientifically valid manner in which to interpret the poor resolution and conformational flexibility when assessing the conformational differences?*

We can see how this might be confusing. However, the differences in the positions of the respective tips of the disulfide arches established by the cryo-EM density maps is on the scale of 10-30 Å, so these differences are clearly resolved even at the worst resolution of 5-7 Å obtained at the very tips. Moreover, much better resolution was obtained along the flanks of the disulfide arches that dictate where the tips are located. Thus, the conformational variability at the tips that is reflected in the local resolution maps is on a much smaller scale than the scale that defines the overall shapes of the arches and the relative positions of the tips. I.e., the lower resolution primarily compromises determination of sidechain orientations rather than the location of the polypeptide backbone. To try to head off the concerns expressed by the reviewer, we have rephrased the sentence on lines 328-331: “Although the available resolution for each of these strains is clearly sufficient to establish that their disulfide arches have distinct overall shapes and relative polypeptide backbone maps, the lower resolution at the tips indicates some local variability in conformation or structure.”

*A second comment on lines 324-328: it's stated that the glycans may impose flexibility to the disulfide arch. but then in the same sentence it's stated that uniform glycans can stabilize the fibril core. This argument requires some additional explanation, as it seems anti-thermodynamic. if the glycans impose additional entropy to the system in the form of conformational flexibility, how can they make it more stable? Then in the next sentence it discusses the inherent instability of the polypeptide core in the arch. please clarify.*

The reviewer raises interesting points, but we simply do not have data or insights to resolve these issues in any more meaningful way, other than to list possible influences on the conformational variability in the tips, even if they seem to be competing or contradictory.

*Very minor points that don't require modification;
Line 365: "This, in turn, might contribute to the well-known strain PrPSc dependent patterns of accumulation and pathological lesions in the brain." and the similar, line 379: "it seems plausible that conformational differences between the strains could modulate how prions interact with other factors". I think the conclusions of this manuscript are strong enough that it's difficult reach any other conclusion". It seems more than just plausible. This is a statement of the 'strain hypothesis', correct?*

We agree with the reviewer, and have strengthened our wording in these sentences as recommended within lines 375-394.

*Paragraph starting line 339: This paragraph contains generalized speculation. The difference in charge distribution between the strains is mentioned multiple times. Trying to read between the lines, do the authors believe that the location and concentration of surface charged residues could lead to divergent biology for the strains? This seems plausible, but leads to the unanswered question as to how that might actually work*.

We see that we could have done better to complete our thought process. Accordingly, we have changed our wording on lines 375-379 as follows: “These types of molecules can differ between cell types or brain regions. Thus, they might interact preferentially with certain prion strains over others due to their respective chemical surfaces and control where within tissues a given strain optimally propagates and accumulates. This, in turn, likely contributes to the well-known strain-dependent patterns of PrP^Sc^ accumulation and pathological lesions in the brain.”

Reviewer #3: *I have minor suggestions.
1) A brief description of fibril variability would be important to include. Was there heterogeneity in fibril structures? Were there any short fibrils examined?*

As suggested, we have added the following description on lines 152-156: “Both individual fibrils of widely varying lengths, and lateral bundles thereof, were observed and those that were not lying on top of one another, regardless of length, were analyzed in the initial 2D images (movies) (Figs. 1 & 2A). Less distinct clumps of unknown nature were also sometimes seen, e.g. in lower right hand corner of Fig. 2A.”

2) Were there differences in the presence, size, and location of non-protein molecules bound to fibrils within a strain, and between strains?

This is an interesting question, for sure, but at present we are only able to say what we have already noted on this issue on lines 200-207: “Similar to the aRML strain (5), and consistent with a22L’s deficiency in N-linked glycans and lack of GPI anchors, we failed to see the peripheral densities adjacent to the attachment sites of these post-translational modifications that had been seen previously with wildtype 263K (6) and RML (7) fibrils (compare Fig. 2D & 3C: open and blue arrowheads). However, consistent with the latter structures, we observed densities adjacent to stacks of cationic sides chains on the surface of the N-terminal half of the core (Fig 2D, orange arrowheads, and Fig. 3F) that may reflect either remnants of more extreme N-terminal PrP sequence after partial proteolysis or non-PrP ligands.”

3) Perhaps the authors could speculate on why the differences in structures are concentrated at the disulfide β-arch.

This is another very interesting and important question, but at this point, we are reluctant to add further speculation to our thoughts expressed on lines 332-342: “All of these fibrils have N-linked glycans within the disulfide arches on at least some of the monomers at N180 or N196, but much more so on the wild-type RML and 263K fibrils. Judging from the relative intensities of the glycosylated versus unglycosylated bands on silver-stained gels, our a22L appears to have a glycan on roughly 1/3 of the monomers. Perhaps such structurally and conformationally diverse glycans (29) impose more flexibility in the disulfide arch even if we and others have shown with molecular dynamics simulations, certain uniform glycans can in theory be accommodated on each rung within a PIRIBS architecture and appear to even stabilize the fibril core (30, 31). Alternatively, there might be more inherent instability in the polypeptide cores in this region of these prion strains that accounts for conformational plasticity. Further studies will be required to which of these factors may be most influential.”

4) The authors may consider further emphasizing the importance of the work to the amyloid field in the Discussion.

A good idea. We have now added the following at the end of the Discussion: “When considered in the larger context of the many diseases caused by the accumulation of pathological protein amyloids, these data reinforce the concept that variations in the core conformations of self-propagating amyloids can underpin marked differences in disease phenotype.”
